# Allogeneic Hematopoietic Cell Transplantation Induces Vessel Wall Inflammation in Large Arteries

**DOI:** 10.1016/j.jacadv.2022.100164

**Published:** 2023-01-11

**Authors:** Sebastian E. Serfling, Wolfgang Thaiss, Anne Wasserloos, Leo Rasche, K. Martin Kortüm, Sabrina Kraus, Takahiro Higuchi, Steven P. Rowe, Malte Kircher, Andreas K. Buck, Hermann Einsele, Ambros J. Beer, Constantin Lapa, Rudolf A. Werner

Allogeneic transplantation is a curative treatment strategy for hematological cancers such as acute myeloid leukemia and is being used as a salvage therapy in patients with multiple myeloma (MM). Due to optimized protocols, transplant-associated mortality and morbidity have been significantly reduced.^1^ However, patients still face cardiovascular events following autologous and allogeneic hematopoietic stem-cell transplantation (HCT), such as acute cardiotoxicity, arrhythmias, onset of heart failure, (non-)fatal myocardial infarction, stroke, or thromboembolism.^2^ Echocardiography in addition to laboratory markers of hemodynamic cardiac stress can be used before transplantation to identify patients at risk of cardiovascular events^2^; however, such screening efforts are limited to detect regional disease activity, including heterogenous inflammatory patterns in the vessels. A whole-body read-out covering disease activity of the entire vasculature is able to overcome this limitation.^3^ In the present study, we hypothesize that increased inflammation in the vessel walls following allogeneic HCT can be detected and monitored by 2-deoxy-2-[^18^F]fluoro-D-glucose ([^18^F]FDG) positron emission tomography (PET)/computed tomography.

We investigated 15 consecutive MM patients who underwent [^18^F]FDG PET/computed tomography before and 3 months after allogeneic HCT. Parts of this cohort have been reported in the article by Stolzenburg et al.^4^ None of the patients had a history of prior vasculitis, anti-inflammatory medication, or thromboembolic cardiovascular events. Previous therapies varied among patients and included novel drugs like proteasome inhibitors, eg, bortezomib, or immunomodulators (lenalidomide).^4^ Providing a noninvasive semiquantitative read-out of the entire vasculature, a total of 8 arteries per patient were analyzed (both carotid arteries, ascending aorta, aortic arch, descending and abdominal aorta, and both iliac arteries). Target-to-background ratios (TBRs), defined as arterial wall uptake divided by unaffected lung uptake serving as reference, were calculated.^3^ TBRs were then correlated with uptake in organs of hematopoietic activation (bone marrow [BM], spleen), as well as C-reactive protein (CRP) levels and white blood cell counts (WBC) as serological markers of systemic inflammation. Baseline and follow-up imaging were investigated, thereby providing the delta TBR between both PETs. We used Pearson’s correlation to determine the association between normally distributed clinical and imaging parameters. The 2-tailed paired or unpaired Student’s *t*-test was used to compare variables between pretherapeutic (TBR_pre_) and post-therapeutic TBR and subgroups with and without cardiovascular risk factors (CVRF, including arterial hypertension, hypercholesterolemia/hyperlipidemia, diabetes, smoking habits, obesity or prior chest radiation). A *P* value of <0.05 was considered significant. The local institutional review board approved this retrospective study.

**Figure 1:**
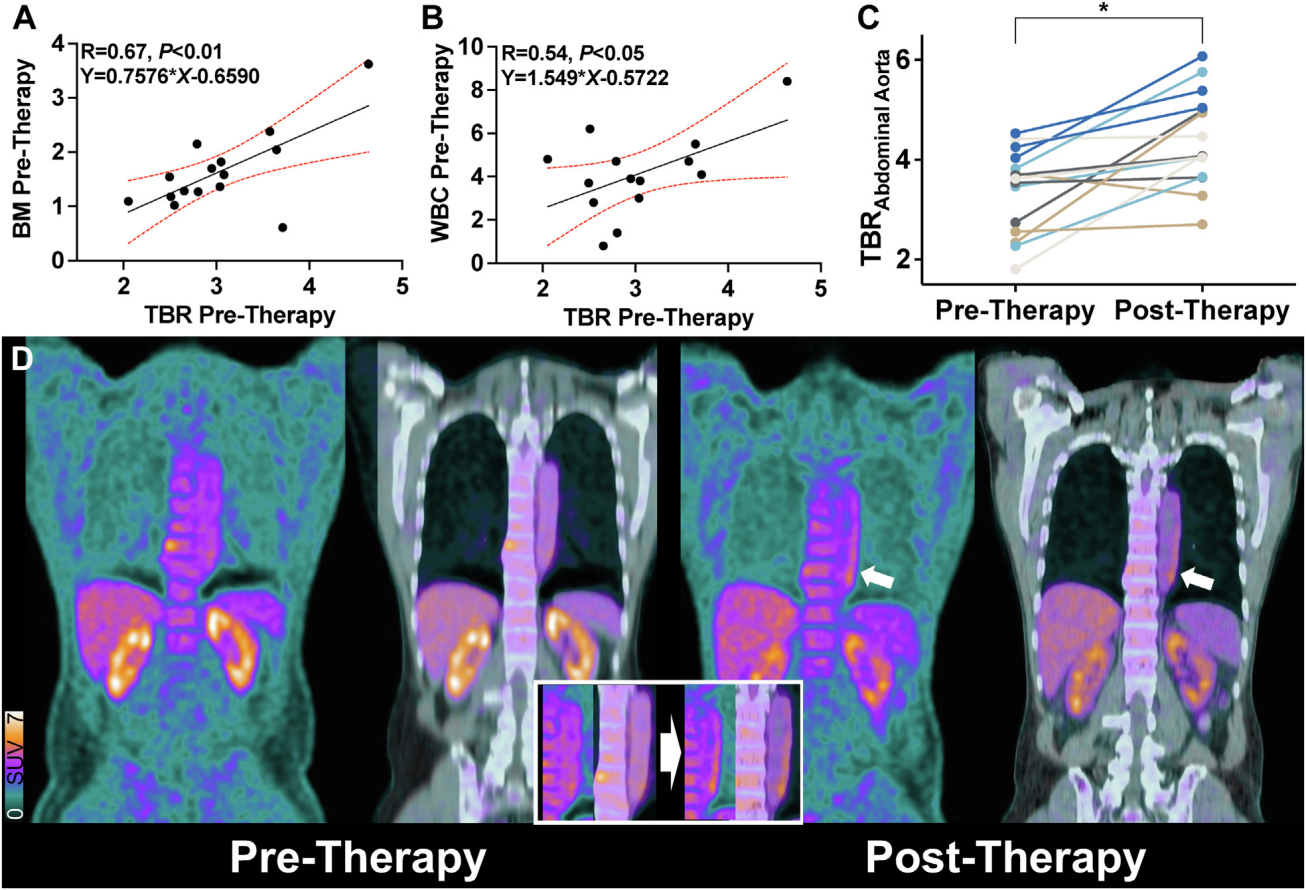
**Inflammatory-Targeted PET Imaging After Transplantation** **(A)** Pretherapeutic uptake derived from bone marrow (BM) and **(B)** white blood cells (WBCs) correlates with pretherapeutic target-to-background ratios (TBRs) derived from all vessel segments (WBC value missing in 1 subject). **(C)** Posttransplant change of TBR (as derived from abdominal aorta) demonstrates an increase in the majority of the subjects. **(D)** Coronal views of positron emission tomography (PET) and PET/computed tomography of the thoracic aorta prior to **(left)** and after **(right)** allogeneic stem cell transplantation. Three months after therapy, vessel wall uptake is markedly increased **(white arrows, inset)**. ∗*P* = 0.001.

For all vessels, the TBR_pre_ was 3.04 ± 0.64 (95% CI: 2.68-3.39) and correlated with uptake derived from BM (R = 0.67; 95% CI: 0.25-0.88; *P* < 0.01) (Figure 1A) and WBC (R = 0.54; 95% CI: 0.011-0.83; *P* < 0.05) (Figure 1B), but not with CRP (R = −0.3; 95% CI: −0.71 to 0.28; *P* = 0.31). Seven of 15 (46.7%) patients presented with CVRF at the time of baseline PET. However, their TBR_pre_ did not significantly differ from those of subjects without CVRF (*P* = 0.08; mean of differences: −0.56; 95% CI: −1.22 to 0.09). After HCT, TBR of all investigated vessel segments increased by 20.1% to 3.64 ± 0.72 (95% CI: 3.24-4.05; *P* = 0.0003 vs TBR_pre_). Those findings were primarily driven by an increase of TBR in the abdominal (30.2%, *P* = 0.001) (Figure 1C) and thoracic aorta (Figure 1D) (aortic arch, 28.4%; ascending aorta, 26%; descending aorta, 18.5%; *P* < 0.05 for all).

The post-therapeutic TBR was not significantly associated with BM activation (R = 0.38; 95% CI: −0.17 to 0.75; *P* = 0.16) or serum markers of inflammation (CRP, R = 0.04; 95% CI: −0.54 to 0.6; WBC, R = −0.01; 95% CI: −0.54 to 0.52; *P* ≥ 0.89, respectively).

Endothelial cell dysfunction syndromes have been reported after allogeneic HCTs as a significant contributor of transplant-associated morbidity.^1^ We are the first to describe a substantial increase of [^18^F]FDG vessel wall uptake, particularly in thoracic and abdominal aortic segments, further supporting the notion that the entire vasculature may be affected after therapy. These findings emphasize the need of an inflammation-directed whole-body molecular imaging approach after allogeneic HCT and may provide a noninvasive biomarker to identify high-risk individuals prone to later cardiovascular events.

Some limitations have to be considered. First, it cannot be ruled out that MM manifestations may act as a glucose reservoir, thereby competing with other tissues such as the vessels for [^18^F]FDG, resulting in altered biodistribution. Thus, after HCT, aortic uptake may be a result of reduced BM uptake from less MM burden. Moreover, 3 months may be too short for an immune system to achieve reconstitution, and thus, future studies may use longer time intervals for follow-up imaging, preferably in a longitudinal setup when the immune response is fully functioning. In addition, the low number of subjects included in our feasibility study did now allow to conduct outcome investigations, eg, to determine the predictive potential of the baseline [^18^F]FDG PET signal for the occurrence of major cardiovascular events after HCT. As such, future studies with larger number of subjects may then allow to provide more robust information on the prognostic value of PET in the context of MM for later cardiovascular outcome.
